# Epidemiological and molecular studies on *Echinococcus granulosus* from free-roaming dogs in Southeast Iran

**DOI:** 10.14202/vetworld.2020.739-745

**Published:** 2020-04-20

**Authors:** Alireza Keyhani, Iraj Sharifi, Mehdi Bamorovat, Mohammad Ali Mohammadi, Asma Askari, Mohammad Ebrahimipour, Majid Fasihi Harandi

**Affiliations:** 1 Student Research Committee, Afzalipour School of Medicine, Kerman University of Medical Sciences, Kerman, Iran; 2 Leishmaniasis Research Center, Kerman University of Medical Sciences, Kerman, Iran; 3 Research Center of Tropical and Infectious Diseases, Kerman University of Medical Sciences, Kerman, Iran; 4 Research Center for Hydatid Disease in Iran, Kerman University of Medical Sciences, Kerman, Iran; 5 Department of Pathobiology, Faculty of Veterinary Medicine, Shahid Bahonar University of Kerman, Kerman, Iran

**Keywords:** echinococcosis, free-roaming dogs, genotype, haplotype network, hydatid cyst, Iran

## Abstract

**Background and Aim:**

Cystic echinococcosis (CE), as a major zoonotic helminth infection, imposes remarkable socioeconomic burden on many endemic countries across the world, including Iran. Due to the high importance of free-roaming dogs in the transmission of CE, epidemiological and molecular studies in this type of hosts are required in the endemic regions. This study aimed to investigate the epidemiology and genotyping of *Echinococcus* *granulosus* isolated from stray dogs in Kerman, Southeast Iran.

**Materials and Methods:**

Eighty-four samples were isolated from stray dogs in the city and suburbs of Kerman in coordination with the health authorities and the municipality office for rabies control and dog population management. Dog demographic data, including age and sex were collected. The worm was isolated by necropsy and genomic DNA was extracted and partial cytochrome c oxidase subunit 1 gene was amplified using specific primers. Phylogenetic and Templeton-Crandall-Sing (TCS) network analyses were carried out on the sequence data.

**Results:**

The overall prevalence of CE in the surveyed dogs was 10.7% (9/84 cases). Out of 84 stray dogs, 33 (39.3%) and 51 (60.7%) cases were male and female, respectively. There was not a statistically significant difference between the infection and gender of dogs. However, infection is shown more in dogs under one year of age with a statistically significant difference (p<0.05). The results of molecular studies indicated *E. granulosus* G1 genotype for all isolates. The high presence of free-roaming dogs in urban and peri-urban areas and high frequency of parasite in this animal is a risk factor for humans in the region. Haplotype sequence analysis on the dog isolates revealed a close relationship with other *E. granulosus* isolates in Kerman.

**Conclusion:**

The findings of this study provide evidence-based data about the epidemiological and molecular characteristics of CE in dog definitive hosts of Southeast Iran. Further studies are required to understand the prevalence and parasite genotypes in dogs in Iran.

## Introduction

Cystic echinococcosis (CE), a major zoonotic helminth infection, imposes a socioeconomic burden in many countries all over the world, including Iran [1-5]. The metacestode of *Echinococcus granulosus* harbors in the viscera of wide variety of herbivores, including sheep, goats, cattle, and camel. The adult worms form in the intestine of dogs and wild canids, including jackals, wolves, and foxes as definitive hosts. These animals have the main role in the distribution of the parasite eggs in the environment. As an endemic region for CE, stray dogs are scattered in large populations among all provinces of Iran. Increasing these dogs, especially around human societies, facilitate the transmission of CE to human and domestic herbivores [6-13]. Molecular studies on the parasite in definitive and intermediate hosts showed ten distinct genotypes (G1-G10) of *E. granulosus* worldwide. Two mitochondrial genes, NADH dehydrogenase 1 and cytochrome c oxidase subunit 1 (CO1) were most frequently used genes in the molecular identification of *E. granulosus* [14-24].

Due to the highly importance of stray dogs in the transmission of CE, epidemiological and molecular studies in these hosts are required in each endemic region for successful implementation of control programs. In Iran, several studies have stressed out on the frequency of *E. granulosus* in dogs and wild canids throughout the country [1,4,6-8]. The mean prevalence of echinococcosis in dogs was 8.8% in Iran, with the range from 8.1% to 11.9% [8-13]. However, little is known on *E. granulosus* genotypes involved in canine echinococcosis in Iran. The molecular studies on isolated parasites from dogs indicated the presence of G1-G3 and G7 genotypes in this important definitive host throughout Iran [25-27]. For the identification of *E. granulosus*, both morphological and molecular techniques are required for providing more exact information about the diversity of parasite in definitive and intermediate hosts.

Due to irrefutable role of stray dogs in the circulation of *E. granulosus* and increasing of their population in the country, more in-depth investigations are required to demonstrate the distribution of echinococcosis in these animals. Studies on the status of echinococcosis in definitive hosts, including dogs, provide evidence-based data to the authorities for better control and management of CE in endemic regions. This study aimed to investigate the prevalence and molecular identification of *E. granulosus* in stray dogs in Kerman, Southeast Iran.

## Materials and Methods

### Ethical approval

This study was undertaken in coordination with the health authorities and also the municipality office for free-roaming dog control. The population size adjustment of stray dogs (in accordance with the WHO guidelines) [28] is applied by these organizations for the control of zoonotic diseases. Samples needed for the current study were isolated from the municipality control unit.

### Study area

This study was performed in the city and suburbs of Kerman, the capital of Kerman Province, Southeast Iran. It is located at 30°17´N and 57° 05´E with 1755 m above the sea level. The city has a hot semi-arid climate and the usual annual precipitation is 135 mm. According to 2016 population census in Iran, the population of Kerman county was 738,724 (statistical center of Iran, 2016). In one study in this region, the population of stray dogs in Kerman Province was estimated to be 145,000-480,000 [29].

### Sampling

**Figure-1:**
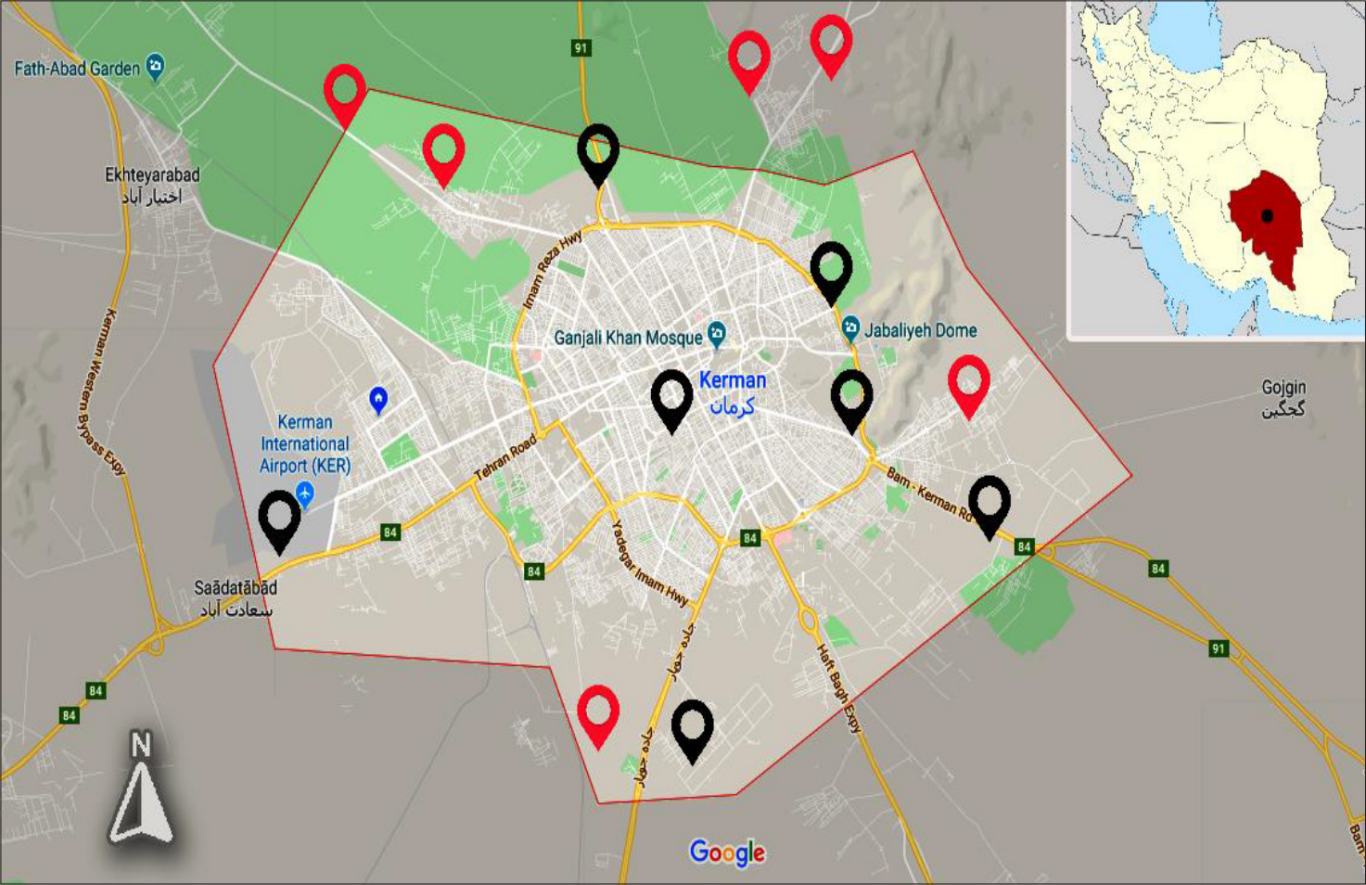
Kerman city map showing the sampling sites where 84 samples from stray dogs were collected. Red pins: Locations where dogs have been found infected with *Echinococcus granulosus*. Black pins: Locations where dogs were found negative [Source: Google maps: https://bit.ly/2wda2rH].

For the months of January to April 2013, 84 samples were isolated from stray dogs in the city and suburbs of Kerman (Figure-1). A questionnaire form was developed and used to collect dog demographic data, including age and sex. The corpse of the stray dogs was opened and the gastrointestinal system was extracted. Afterward, the intestine was cut into two segments, including small and large intestine. In the laboratory, the small intestine was opened and scrapped entirely and contents were washed through a fine sieve into a suitable container. The filtrate retained in the sieve was washed into a plate and surveyed carefully for the existence of *E. granulosus*. For diagnosis, the collected worms were suspended in 70% ethanol. The parasites were identified according to the standard helminthology keys [30,31]. The severity of infection in the stray dogs was determined as light (1-200), medium (201-1000), and heavy (>1001), according to Macpherson *et al*. [32].

### Molecular identification and phylogenic analysis

Genomic DNA of five randomly selected worms from each infected dog was extracted by Accua Prep DNA extraction kit (Bioneer, South Korea) and partial region of CO1 gene was amplified using specific primers [33]. Polymerase chain reaction (PCR) products were electrophoresed in 1% agarose gels and visualized in ultraviolet transilluminator. The purification of PCR products has been done with specific kit (Macrogen, South Korea) and was sequenced for haplotype identification. All sequences were compared pairwise with each other and also with deposited sequences in GenBank using program nucleotide BLAST of the NCBI (http://blast.ncbi.nlm.nih.gov/Blast.cgi). Equal length for each sequence was trimmed with Bioedit software v.7.2 (http://www.mbio.ncsu.edu/BioEdit/bioedit.html) and global sequence alignments were performed using ClustalW algorithm. A phylogenetic analysis was carried out on the sequence data obtained in the present study and the data were compared with other species/genotypes of *Echinococcus*. The best-fit nucleotide substitution model and phylogenetic tree were generated using Mega 6 software (https://www.megasoftware.net). The reliability of the obtained tree topologies was tested with 1000 bootstrapping replicates. In addition, all available mitochondrial COI records of *Echinococcus* isolates of human and animal origin from Kerman were collected from NCBI and a haplotype network analysis was carried out by Population Analysis with Reticulate Trees (PopART) software (http://popart.otago.ac.nz) using statistical parsimony with 1000 times iterate [34-36].

### Statistical analysis

Descriptive and analytical statistics were done for collected data. Primary screening was performed using two K contingency tables (cross-tab) of exposure variables by Chi-square and Fisher exact tests. All data were analyzed using SPSS software (version 20.0, SPSS, Inc., Chicago, IL, USA) and p<0.05 was considered as statistically significant.

## Results

### Epidemiological survey

The overall prevalence of *E. granulosus* infection in surveyed dogs was 10.7% (9/84 cases). Out of 84 stray dogs, 33 (39.3%) and 51 (60.7%) cases were male and female, respectively. The adult worms were mostly isolated from jejunum and ileum. The parasite burden categorization revealed seven and two infected dogs as moderately and heavily infected with gravid adult worms, respectively. More than half of the stray dogs were <3 years old. The frequency of dogs in different age groups is shown in Table-1. There was not a statistically significant difference between having infection and gender of dogs. However, there was a statistically significant difference between echinococcosis and age (p<0.05) of dogs and infection is shown more in dogs under one year of age.

**Table-1:** Age and sex distribution of *E. granulosus* infection of stray dogs in the city and suburbs of Kerman, Kerman Province, Southeast of Iran, 2012-2013.

Characteristics	No. Infected (%)	No. not infected (%)	Total (%)
Age (year)			
<1	6 (7.14)	20 (23.80)	26(31.30)
1-3	2 (2.38)	24 (28.57)	26(31.30)
3-6	1 (1.19)	21(25)	22(26.30)
>6	0 (0)	10 (11.90)	10(11.30)
Gender			
Male	4 (4.76)	29 (34.52)	33 (38.75)
Female	5 (5.95)	46 (54.76)	51 (61.25)
Total	9 (10.71)	75 (89.29)	84 (100)

E. granulosus=Echinococcus granulosus

### Molecular study

**Figure-2:**
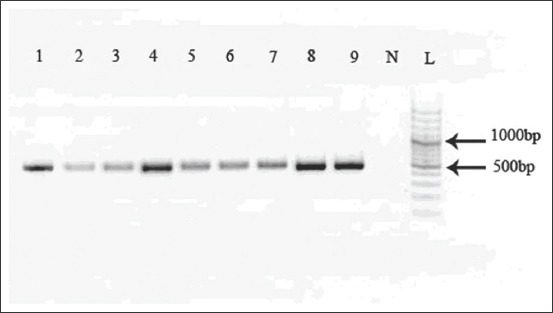
Polymerase chain reaction (PCR) products of 444 bp of the partial region of cytochrome c oxidase subunit 1 gene. PCR products for each extracted DNA were separated by electrophoresis on 1% agarose gels. Number (1-9) positive samples, (N) no templet control, and (L) ladder.

**Figure-3:**
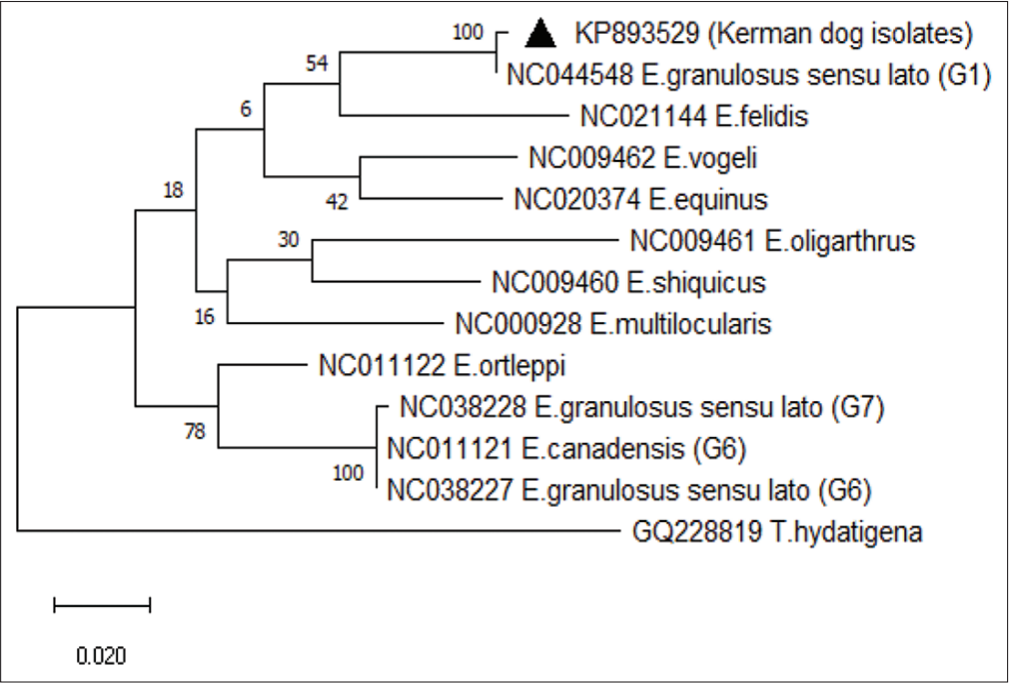
Molecular phylogenetic tree of mitochondrial CO1 region of *Echinococcus* *granulosus* isolates from Kerman stray dogs. Phylogenic analysis was done based on partial CO1 gene sequences (366 bp) data obtained in the present study and other RefSeq data from other species/genotypes of *Echinococcus* using maximum likelihood method based on the kimura2 parameter model with MEGA 6 software.

**Figure-4:**
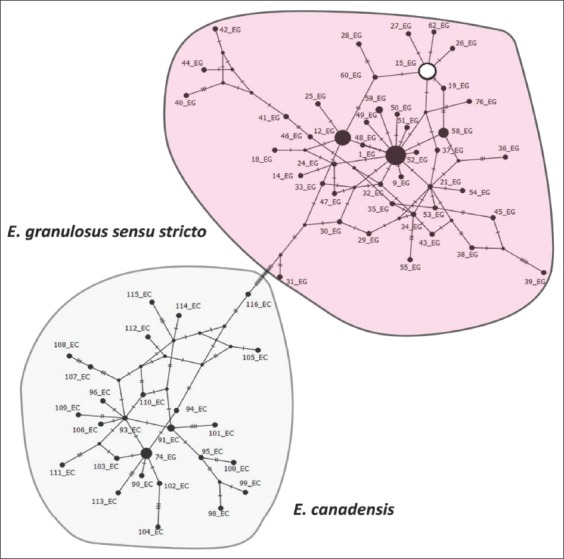
Network analysis of mitochondrial cytochrome c oxidase subunit 1 (CO1) haplotypes of all available mitochondrial CO1 records of Echinococcus isolates of human and animal origin from Kerman collected from NCBI. The analysis was carried out by Population Analysis with Reticulate Trees (PopART) software using statistical parsimony with 1000 times iterate. The size of circles indicates the frequency of the haplotypes. The circles are identified by the corresponding accession numbers. Empty circles represent haplotypes detected in the current study.

PCR amplification of 444 bp of the partial region of CO1 gene was amplified for each extracted DNA (Figure-2). Molecular studies and sequencing of PCR products identified all samples as *E. granulosus* G1 genotype. The obtained sequence in the current study was directly submitted into GenBank under the accession number, KP893529. Phylogenetic analysis showed a close relationship among isolated worms and other haplotype records of *Echinococcus* species in Kerman Province (Figure-3). A haplotype network of CO1 gene diversity in representative Kerman *E. granulosus* isolates is shown in Figure-4. The sequences of the isolated worms from dogs were found closely related to other *E. granulosus* isolates in Kerman.

## Discussion

Such as most endemic countries, the precise status of CE in intermediate and definitive hosts still remains unclear in Iran [12,35]. As the main definitive host for CE, more in-depth investigations on the epidemiology of infection in dogs are required in endemic countries. To the best of our knowledge, for the first time, the epidemiological study powered by molecular characterization on isolated *E. granulosus* form stray dogs in Kerman Province was conducted in the current study [1,3,37].

The high prevalence (10.71%) of CE in the current study emphasizes the importance of stray dogs in the survival of parasite in the environment. Many studies in Iran were conducted on the epidemiology of echinococcosis in dogs and the mean prevalence of CE in these important definitive hosts showed 8.8% with the range from 8.1% to 11.9%. It was found that the infection is more in dogs below 1-year age with a statistically significant difference (p<0.05). In line with the current study, Tackmann *et al*. [38] demonstrated a higher prevalence of CE in juvenile dogs. A high prevalence of CE in these younger dogs has been shown in the previous study in Iran with 41.67% infection in dogs below 1-year age [8]. This high infection in younger dogs (<1 year) could be due to incomplete development of their immune system. In contrast with the previous study in Iran [8], female dogs were more infected with *E. granulosus* but without any statistically significant difference. Regardless of age and gender of dogs, these animals are infected with *E. granulosus* without any clinical symptoms and they left untreated in nature. The high presence of stray dogs in urban and peri-urban areas and high frequency of parasite in this animal is a risk factor for human CE in Iran.

Due to differences in the prevalence, longevity, morphology, period of egg production, host specificity, geographical distribution, and pathogenicity of various genotypes of *E. granulosus*, genotyping to identify this parasite is important for evidence-based control and the prevention of CE in each endemic region. According to the previous studies on *E. granulosus* genotypes in Iran, the presence of G1, G2, G3, G5, G6, and G7 genotypes was characterized in different intermediate and definitive hosts all over the country [4,11,17,19,27,39-47]. All isolated parasites in the current study were molecularly characterized as G1 genotype of *E. granulosus*. Phylogenetic study along with TCS network haplotype analysis was shown a close relationship between the *Echinococcus* isolates in the present study and the previous haplotype records of *Echinococcus* in Kerman Province. Many molecular investigations on genotype identification of isolated *E. granulosus* from dogs were done and it was indicated the presence of G1-G3, G6, and G7 genotypes in this important definitive host in Iran [11,48,49]. In one study performed in West Iran, genotyping analysis of 71 isolated parasite showed the presence of G1 (75%), G2 (10%), and G3 (15%) genotypes in dogs [27]. Two studies in China and India showed naturally infected dogs with G1 as main genotype in these endemic regions [50,51]. Due to most frequency of G1 genotype in human CE in Iran, the high prevalence of this genotype in dogs should be more considered for public health [52,53]. Increasing burden of CE in dog definitive host can seriously face humans at risk of CE [54]. The role of dogs in the transmission of parasite has not been clarified for people in endemic regions. Inadequate education about lifecycle of the parasite in dog owners, butchers, and abattoir workers is the main obstacle in the control of CE in endemic countries, including Iran [55]. Dog dosing should be routinely implemented in endemic countries for declining worm burden in these important hosts.

## Conclusion

The findings of this study provide evidence-based data about the epidemiological and molecular characteristics of CE in dogs as a definitive host in Kerman, Southeast Iran. For effective control of CE, new strategies and investigates should be designed in this endemic region. Further studies are required to understand the prevalence and parasite genotypes in dogs in Iran.

## Authors’ Contributions

MFH, MB and AK: Conceptualization. MFH, MAM, and AK: Data curation and formal analysis. MFH: Funding acquisition. MFH, AK, AA, MB, and MAM: Investigation. MFH, IS, and ME: Project administration.MFH and AK: Resources. MFH, AK, and MAM: Software. MFH, AK, MB, and MAM: Writing original draft. MFH, ME, MB and IS: Writing review and editing. All authors read and approved the final manuscript.
